# Caveolin-1 overexpression predicts poor disease-free survival of patients with clinically confined renal cell carcinoma

**DOI:** 10.1038/sj.bjc.6601359

**Published:** 2003-11-11

**Authors:** L Campbell, M Gumbleton, D F R Griffiths

**Affiliations:** 1 Pharmaceutical Cell Biology, Welsh School of Pharmacy, Cardiff University, Cardiff, CF10 3XF, UK; 2 Department of Pathology, University Hospital of Wales, Cardiff, CF14 4XN, UK

**Keywords:** Caveolin, renal cell carcinoma, vascular invasion, metastasis, prognostic indices

## Abstract

Renal cell carcinomas, although usually apparently fully resected at surgery, commonly recur as distant metastasis. New markers are needed to predict which patients may relapse especially as novel methods of treatment (e.g. laproscopic resection) may make it impossible to assess conventional pathological prognostic markers. The caveolins are a family of proteins that represent the major structural components of caveolae; recent work suggests that these may have influence on several signalling pathways and they are thus potential prognostic markers. Immunohistochemistry for caveolin-1 was performed on sections of peripheral tumour from 114 consecutative nonmetastatic RCCs. Cytoplasmic caveolin-1 immunohistochemical (ICC) reaction was scored on a semiquantative scale of 1–3. Immunohistochemical score was tested for impact on disease-free survival by Kaplan–Meier and Cox regression methods. A total of 50 tumours had ICC score 1; 43 had score 2 and 21 score 3. Larger, higher grade and tumours with vascular invasion had significantly higher scores. On univariate survival analysis (Kaplan–Meier), patients with tumours scoring 1 had a mean disease-free survival of 6.61 years (95% CI 5.76–7.46) compared with 5.4 years (4.53–6.30) and 3.15 years (1.87–4.44) for scores 2 and 3, respectively. This is a significant difference (*P*=0.0017 log rank test). On multivariate analysis with size, grade and caveolin ICC score as independent covariates, caveolin ICC score 3 was an influential predictor of poor disease-free survival with a hazard ratio of 2.6 (*P*=0.03). We conclude that cytoplasmic overexpression of caveolin-1 predicts a poor prognosis in RCC; that this is likely to be a useful prognostic marker and that it may have importance in tumour progression.

Renal cell carcinoma (RCC) is the most common malignancy of the kidney and is responsible for 3% of all cancers in adults and about 2% of cancer-related deaths (Dhote *et al*, 2000). At presentation, about two-thirds of RCCs are clinically localised and as a result such patients will usually undergo nephrectomy. Unfortunately, following surgical resection, approximately 40% of patients treated for apparently localised disease will relapse and die as a result of distant metastasis (Ljungberg *et al*, 1999). Assessment of the risk of developing subsequent metastatic disease is notoriously difficult, and currently accepted prognostic indicators are recognised as not offering the accuracy required for confident prognostication and selection of patients for further therapy. It is clear that the identification of additional prognostic markers will be of benefit to the selection of high-risk patients for adjuvant therapy, particularly as novel methods of treatment, for example laproscopic resection may, by morcellating the resected tumour, make it impossible to determine all conventional pathological prognostic parameters (Rabban *et al*, 2001).

The caveolins are an evolutionary conserved family of proteins that represent the major structural and functional components of caveolae, which are specialised invaginated lipid microdomains present at the plasma membrane of most mammalian cells (Razani *et al*, 2002). Caveolin-1 was the first of the caveolin supergene family to be discovered over a decade ago and as a result remains the most extensively studied. Caveolin-1 was first identified as a novel substrate for the src kinase oncogene in virally transformed fibroblasts (Glenney, 1989). Since then several functions have been ascribed to caveolae and caveolin, including the compartmentalisation of members of numerous signalling cascades. This allows crosstalk between distinct linear signalling pathways and the negative regulation of the kinase activity of several key signalling molecules. Among others, these include the epidermal and platelet growth factor receptors, p42/p44 MAP kinases, heterotrimeric G-proteins, Src family tyrosine family kinases and nitric oxide synthases (Shaul and Anderson, 1998). Additionally, the expression of recombinant caveolin-1 is sufficient to restrict the growth potential of transformed cells isolated from primary tumours of the breast (Lee *et al*, 1998), lung (Racine *et al*, 1999) and ovaries (Bagnoli *et al*, 2000). In this regard, caveolin-1 is proposed to represent a novel tumour suppressor protein. Consistent with this the chromosomal loci of caveolin-1 and -2 has been recently identified as 7q31.1 (Engelman *et al*, 1999), a fragile site frequently deleted in a wide spectrum of human cancers that includes cases of sporadic RCC. Such chromosomal rearrangements may result in translocations, deletions or amplification of genes at this locus (Shridhar *et al*, 1997), although the functional significance of such changes on caveolin in RCC remain to be determined.

However, much of the current literature that lends support for caveolin-1 serving as a tumour suppressor protein has been generated by the use of *in vitro* and *ex vivo* overexpression systems for caveolin-1 that are assumed to mimic the activity of endogenously expressed caveolin. To this end a recent series of clinicopathological studies have determined that the upregulation of caveolin-1 is indeed associated with tumour progression and/or poor prognosis in several distinct cancers such as adenocarcinomas of the prostate (Yang *et al*, 1999), lung (Ho *et al*, 2002), pancreas (Ho *et al*, 2002) and squamous cell carcinoma of the oesophagous (Ho *et al*, 2002).

There are no previous studies of renal cancer and caveolin. We have therefore designed and carried out a study in which we examined the immunohistochemical (ICC) detected expression of caveolin-1 in 114 RCCs from patients treated for localised disease, and assessed its impact on disease-free survival. The aim of the study is to determine if caveolin-1 expression might have value as a prognostic marker and if it has a potential role in tumour progression.

## PATIENTS AND METHODS

### Patient selection

The tumours studied were a consecutive series of RCCs identified from the pathological records of one hospital. This is a subset (from one hospital) of a series that has been reported in detail previously (Griffiths *et al*, 2002). All patients had undergone surgery for a primary kidney tumour and none had evidence of either lymph node or distant metastatic disease either before or at surgery. Histology reports and slides were available in all cases and all included at least two blocks of the edge of the tumour and a block of renal vessels. Each tumour slide was reviewed by a pathologist without knowledge of the clinical outcome and assessed for histological type by the Heidelberg classification (Kovacs *et al*, 1997); for Fuhrman nuclear grade (Fuhrman *et al*, 1982); the presence or absence of any vascular invasion (either microvascular invasion, renal vein invasion or inferior vena cava invasion); and whether or not there was capsular penetration with cellular invasion of perinephric fat (Thomas *et al*, 2003).

The median age of the 114 patients was 63.6 years (range 33–84); 76 were men and 38 women; 69 of the tumours were clear cell carcinomas, 17 papillary carcinomas, four chromophobe carcinomas and there was one collecting duct carcinoma. A total of 23 of the tumours were unclassified by conventional histology. Most of the patients had been reviewed annually as outpatients for between 3 and 7 years. The following information was obtained from the patients notes: date of birth, sex, date of surgery, date last seen, date of death, cause of death and the date on which recurrent or metastatic disease was first identified. Two cases were lost to follow-up at 25 and 29 months. In all other survivors, the last recorded clinical contact was after 1 January 98, giving a median follow-up of 44 months (range 1–99). In nine patients in whom the cause of death was recorded as renal cell cancer, the date of first recurrence was not available; in these cases, the date of death was considered the end point for disease-free survival.

Paraffin blocks were available in all cases, and for each renal carcinoma a block was selected that contained a sample of peripheral tumour. Sections were cut onto cleaned slides (Superfrost Plus™) at 4 *μ*m thickness.

### Antibodies and reagents

The rabbit polyclonal anti-caveolin-1 was obtained from BD Transduction Laboratories (Oxford, UK). This antibody recognises both the *α* and *β* isoforms of caveolin-1 as assessed by Western blotting. The secondary swine anti-rabbit horseradish peroxidase (HRP)-conjugated antibody, as well as nonimmune rabbit serum were obtained from DAKO (Cambridge, UK)

### Immunohistochemistry

Immunohistochemical staining for caveolin-1 was carried out using standard methodologies as previously described (Campbell *et al*, 2002). Briefly, following removal of paraffin wax, endogenous peroxidase activity within the rehydrated sections was blocked with 0.6% hydrogen peroxide in methanol for 15 min at room temperature. The slides were then briefly washed in tap water before each section was equilibrated in Optimax™ wash buffer (pH 7.4; Menerium Diagnostics, Oxford UK) at room temperature for an additional 10 min. After draining, the primary antibody was applied to each section at a dilution of 1 : 10 (diluent was 0.6% BSA in Optimax™ wash buffer) and incubated overnight at 4°C for a total of 15 h. The next day, sections were washed (4 × 1 min) with Optimax™ buffer and the appropriate secondary HRP-conjugated antibody was applied at a dilution of 1 : 100 for 1 h at room temperature. Following this further washes were undertaken to remove any unbound reporter antibody, and immunoreactivity was subsequently detected using the 3,3′-diaminobenzidine system (Sigma, Poole, Dorset, UK). The sections were counterstained with haematoxylin and finally mounted.

### Controls and scoring of stained specimens

Negative controls run in parallel comprised sections where the primary antibody had been omitted or replaced with nonimmune rabbit serum. Caveolin-1 staining of peripheral endothelial cells and nephric fat within the sections was monitored and served as positive internal control stain for immunoreactivity.

Scoring of sections was performed by a pathologist (DFRG) and a research associate (LC). Caveolin-1 staining was assessed by light microscopy using a double-headed microscope without knowledge of the clinical outcome, and each tumour was allocated a score by consensus in a semiquantitative way against tabulated criteria as follows: 0: no detectable deposit in tumour cells; 1: very light diffuse or focal light deposit in tumour cell cytoplasm; 2: light diffuse or moderate focal deposit (but may include very small areas of heavy deposit); 3: tumour containing areas of heavy deposit in tumour cells. Only two tumours scored zero and these cases were pooled with score 1 for subsequent analysis.

### Statistics

Correlations between variables were examined by crosstabulation and *χ*^2^ testing and Spearman correlation as appropriate. For disease-free survival, deaths due to causes other than RCC were considered to be censored at the date of death. Univariate analysis of disease-free survival was carried out by the Kaplan–Meyer method using the Log rank test. Multivariate survival analysis was carried out by Cox regression; in the case of grade, capsular invasion, vascular invasion and caveolin-1 score, a linear contrast was employed such that comparison was made with grade 1, caveolin score of 1, and the absence of vascular and capsular invasion, respectively. Initially, all covariates were entered into the model; covariates for the final model were selected by a forward conditional method with the probability of entry of 0.10 and of rejection of 0.15. A second Cox regression analysis was carried out entering only the variables that would be available following a laproscopic resection in which the tumour had been morcellated, that is, excluding capsular invasion and vascular invasion. All statistical analysis was carried out using the program SPSS for Windows 9.0, (SPSS Inc, Chicago, IL, USA). Where appropriate, all tests of significance were two-tailed.

## RESULTS

In normal renal parenchyma (adjacent to some of the tumours), the strongest ICC staining for caveolin-1 was localised to proximal tubular epithelium cytoplasm in a fine granular pattern. Similar but weaker staining was seen in distal tubules, the medullary tubules and capsular glomerular epithelium. Strong staining was also noted in the endothelium of arteries, arterioles and the vasa recta. However, no staining was seen in the podocytes, the glomerular capillaries or the peritubular capillaries. There was consistently strong staining of the cell membrane of the adipocytes in the renal sinus.

**Figure 1:**
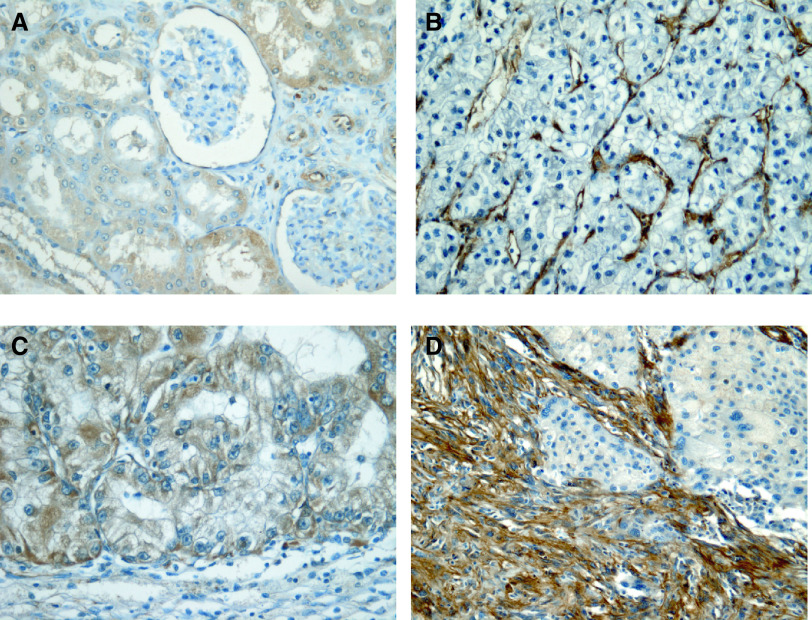
Caveolin-1 immunohistochemistry of kidney and renal tumours. (**A**) Normal kidney showing granular immunoreactivity in the proximal tubular cells endothelium of arterioles and capsular epithelium; (**B**) RCC with score 1, note the strong vascular immunoreactivity; (**C**) RCC with caveolin score 2 and (**D**) high-grade sarcomatoid RCC in the lower left part of the image with caveolin score 3.

Two tumours, both well-differentiated conventional RCCs, showed predominant cell membrane staining. This was not seen in others and was ignored for the purposes of this study. In the remainder, the localisation and the nature of staining, if present , was granular and cytoplasmic and qualitatively similar to that seen in the normal proximal tubule (Figure 1

). In all, 50 cases were scored for staining intensity semiquantativly as 0 or 1; 43 cases as score 2 and 21 as score 3. In practice, tumours with score 3 had substantial areas of tumour cells with heavy cytoplasmic deposit. The crosstabulation of the scores and the other prognostic determinants is shown in Table 1

**Table 1:** Crosstabulation of prognostic indices and caveolin-1 score

	**Caveolin-1 score**				
	**1.0**	**2.0**	**3.0**	**Total**	
**Prognostic indices**	***n*=50**	***n*=43**	***n*=21**	***n*=114**	**Statistics**
Tumour Grade 1 and 2	37	33	9	79	*P*=0.008
Tumour Grade 3	13	6	9	28	
Tumour Grade 4		4	3	7	
Tumour size <5 cm	16	11	4	31	*P*=0.133
Tumour size 5–7 cm	15	12	2	29	
Tumour size >7 cm	19	20	15	53	
Vascular Invasion (−ve)	36	24	6	66	*P*=0.003
Vascular Invasion (+ve)	14	19	15	48	
Capsular Invasion (−ve)	43	35	15	93	*P*=0.352
Capsular Invasion (+ve)	7	8	6	21	

. More intense caveolin staining was found significantly more frequently in high-grade tumours, and in tumours showing any vascular invasion (Pearson's *χ*^2^ *P*=0.008 and 0.003, respectively). When analysed for level of vascular invasion, there was a significant trend to increasing caveolin-1 expression with more advanced vascular invasion (Table 2

**Table 2:** Level of vascular invasion crosstabulated with caveolin score

	**Caveolin score o or 1**	**Caveolin score 2**	**Caveolin score 3**	
Microvascular invasion	9	5	4	Spearman correlation *P*=0.038
Renal vein invasion	3	8	5	
IVC invasion	2	6	6	

).

**Figure 2:**
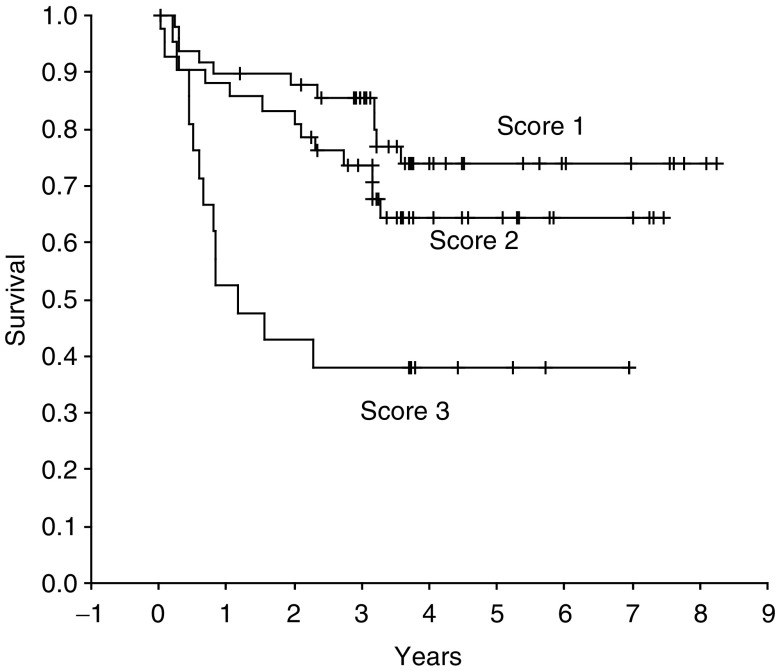
Kaplan–Meier disease-free survival plot of calveolin score (*P*=0.0017 log rank test).

Survival analysis showed that tumours with more caveolin staining had shorter time to tumour recurrence. On univariate survival analysis (Kaplan–Meier), patients with tumours scoring 1 had a mean disease-free survival of 6.61 years (95% CI 5.76–7.46) compared with 5.4 years (4.53–6.30) and 3.15 years (1.87–4.44) for scores 2 and 3, respectively (Figure 2

). This is a significant difference (*P*=0.0017 log rank test). On Cox regression analysis, including covariates tumour type, size, vascular invasion, capsular invasion grade and caveolin score, the influential covariates predicting relapse were grade, capsular invasion and vascular invasion; the covariates caveolin score, tumour size and tumour type being rejected by the selection criteria. However, in a Cox regression model with the covariates size, grade, caveolin score and tumour type, caveolin score is a significant influential covariate together with tumour size and grade (Table 3

**Table 3:** Cox regression analysis including tumour type (rejected by selection criteria) tumour size, tumour grade and caveolin score

	**Hazard ratio**	**95% CI**	***P***
Grade 1 and 2	1		
Grade 3	1.3	0.6–2.9	0.47
Grade 4	6	2.3–16.0	0.0003
Caveolin score 0 and 1	1		
Cavolin score 2	1.4	0.6–3.2	0.43
Caveolin score 3	2.6	1.1–6.2	0.03
Size^a^	1.17	1.05–1.29	0.003

a Hazard ratio is for each increase in size by 1 cm.

).

## DISCUSSION

We have shown that caveolin-1 overexpression, as determined by immunocyochemistry, is an important prognostic determinant in RCC. This is the first demonstration that caveolin-1 expression correlates with outcome in RCC; this observation requires a biological explanation and provides the opportunity to consider potential clinical applications as a prognostic marker.

The relationship between positive caveolin-1 expression within this particular patient cohort and other excepted clinicopathological prognostic variables currently used in RCC is similar to other pathological findings conducted on other tumour types. For example, in prostate cancer, increased caveolin-1 expression correlates with Gleason score, positive surgical margins, lymph node metastasis and androgen insensitivity (Yang *et al*, 1999). Additionally, caveolin-1 overexpression is related to tumour size and histopathological stage in both pancreatic ductal carcinoma (Suzuoki *et al*, 2002) and oesphageal squamous cell carcinoma (Kato *et al*, 2002). In all instances, elevated caveolin- 1 levels were associated with tumour progression and poor prognosis.

To date little is known about the precise function of caveolin-1 in the normal functioning of kidney, but it has been shown to be present in collecting ducts (Tajika *et al*, 2002) and proximal tubule cells (Edwards, 1999) of rat kidney, where it is shown to be localised with the water protein channel aquaporin-2 and the chloride channel huH1, respectively. These observations suggest a role for caveolin-1 in the regulation of water reabsorption. In mice, it has been colocalised with a calcium ATPase pump within the plasma membrane of distal tubular cells where evidence implicates a function related to calcium reabsorption (Cao *et al*, 2003). Our findings suggest normal cytoplasmic localisation of caveolin-1 in the proximal and distal tubules and capsular glomerular epithelium in normal human kidney, but no apparent expression was observed in podocytes, glomerular capillaries or peritubular capillaries. However, our technique may not be sensitive enough to identify low levels of membrane expression. Apparent from the results of our current study was the cytoplasmic localisation of the overexpressed caveolin-1 within the renal carcinoma cells. Such a subcellular location as opposed to its more traditional position at the plasma membrane appears to be a prevailing feature within different types of human cancer where caveolin-1 levels are elevated as is the case for tumours of the colon (Fine *et al*, 2001), pancreas (Suzuoki *et al*, 2002) and prostate (Yang *et al*, 1999). This suggests that, within the transformed cells, caveolin-1 is rerouted into the secretory pathway of these cells, and that such an inappropriate caveolin-1 localisation and/or accumulation may contribute to the transformed phenotype. Indeed within prostate cancer cell lines, caveolin-1 has been shown to be secreted in response to androgens and glucocorticoids. This secreted caveolin is sufficient to permit survival and clonal growth of these cells, thereby contributing to their metastatic potential and androgen insensitivity (Tahir *et al*, 2001). It is not clear if similar mechanisms occur within the context of RCC. However, from what is currently known from other studies, caveolin-1 may serve as an important intercellular signalling molecule that is capable of potentiating the progression, invasiveness and vascularisation of renal tumours. In support of this hypothesis, caveolin-1 is shown to interact and potentiate the activity of metalloproteinases (Puyraimond *et al*, 2001) and the urokinase receptor (Stahl and Mueller, 1995; Wei *et al*, 1999) in a variety of cell types, leading to activation of cell surface plasminogen, and generation of plasmin, which in turn leads to the overall degradation of extracellular matrices. Recently, Lisanti and co-workers (Liu *et al*, 2002) have shown that, within an *in vitro* model of angiogenesis, the direct delivery of caveolin-1-derived peptides to the cytoplasm of endothelial cells is sufficient to induce capillary tubule formation. These findings are of significance since several independent studies advocate that the presentation of angiogenesis and microvascular invasion in patients who undergo surgery for RCC are at high risk for developing metastatic disease (Van Poppel *et al*, 1997; Dekel *et al*, 2002; Griffiths *et al*, 2002). More work will be required to determine whether or not caveolin-1 immunoreactivity is a useful clinical marker. Our series is small, but nevertheless suggests that caveolin-1 immunoreactivety is an impressive predictor of poor disease-free survival. It will be important to determine if this can be confirmed in other series, and if the effect is similar in all tumour subtypes. With more cases it may be possible to determine if caveolin-1 expression is an influential covariate when all other known prognostic determinants are included in the Cox model. Even if this is not the case, our analysis shows that when vascular invasion and capsular invasion are excluded, the hazard of caveolin score 3 is 2.6 times that of caveolin score 1. This is similar to the hazard of the presence of vascular invasion, an established and robust prognostic determinant. It is also noted that caveolin-1 immunoreactivity positively correlates with vascular invasion; our results therefore suggest that caveolin immunoreactivity is likely to be of value in assessing prognosis when it is not possible to assess vascular invasion. An example of the latter would include laparoscopic nephrectomy for the resection of renal tumours via a small incision in the abdominal wall. Given that these incisions are typically 10–12 mm in diameter, the removal of intact tumours in some instances is impossible; this therefore requires *in situ* morcellation, rendering assessment of vascular invasion difficult (Rabban *et al*, 2001). Determination of the degree of caveolin-1 expression in these tumour fragments may be of additional value in the staging of renal tumours and assessment of prognosis.

In summary, we have shown that elevated immunoexpression of caveolin-1 is a predictor of poor disease-free survival in RCC, suggesting that cell signalling pathways involving caveolin-1 may have importance in tumour progression. Furthermore, the strength of the association with poor prognosis and its association with vascular invasion suggest a role in assessing prognosis for clinical use.
